# Intrusion Detection of UAVs Based on the Deep Belief Network Optimized by PSO

**DOI:** 10.3390/s19245529

**Published:** 2019-12-14

**Authors:** Xiaopeng Tan, Shaojing Su, Zhen Zuo, Xiaojun Guo, Xiaoyong Sun

**Affiliations:** College of Artificial Intelligence, National University of Defense Technology, Changsha 410073, China; tanxiaopeng14@nudt.edu.cn (X.T.); susj-5@163.com (S.S.); jeanakin@nudt.edu.cn (X.G.); sxy15084973192@163.com (X.S.)

**Keywords:** deep belief network, PSO, parameter optimization, intrusion detection

## Abstract

With the rapid development of information technology, the problem of the network security of unmanned aerial vehicles (UAVs) has become increasingly prominent. In order to solve the intrusion detection problem of massive, high-dimensional, and nonlinear data, this paper proposes an intrusion detection method based on the deep belief network (DBN) optimized by particle swarm optimization (PSO). First, a classification model based on the DBN is constructed, and the PSO algorithm is then used to optimize the number of hidden layer nodes of the DBN, to obtain the optimal DBN structure. The simulations are conducted on a benchmark intrusion dataset, and the results show that the accuracy of the DBN-PSO algorithm reaches 92.44%, which is higher than those of the support vector machine (SVM), artificial neural network (ANN), deep neural network (DNN), and Adaboost. It can be seen from comparative experiments that the optimization effect of PSO is better than those of the genetic algorithm, simulated annealing algorithm, and Bayesian optimization algorithm. The method of PSO-DBN provides an effective solution to the problem of intrusion detection of UAV networks.

## 1. Introduction

In recent years, with the rapid development of cloud computing and artificial intelligence technology, the Internet of Things technology has also ushered in vigorous development. Various intelligent devices can receive a large amount of information through data exchange and interconnection. The popularity of the Internet of Things technology and the intelligence of devices have brought great convenience to people, but the use of new technologies and smart devices has also brought new security and privacy risks [1,2,3]. As the Internet of Things nodes collect and store large amounts of user privacy data, Internet of Things systems have become important targets for cyber attackers. In this case, protecting personal privacy and data security is very important [4,5,6].

With the progress of technology and the continuous reduction in manufacturing costs, the Internet of Things system composed of unmanned aerial vehicles (UAVs) has entered industrial production and people’s daily life from the military field. Nowadays, UAVs have been widely used in film and television shooting, agricultural monitoring, meteorological monitoring, forest fire detection, emergency rescue, and other fields. However, while UAVs bring various conveniences to our production and life, the network security problems they face have been gradually exposed [7,8].

When multiple UAVs cooperate to perform tasks, it is necessary to build information connection channels between them to form a mobile self-organizing network of UAVs. The UAVs in the network realize the real-time sharing of information through this mobile network, which no longer needs to be forwarded by a ground station, and this effectively improves the survivability and combat ability of the UAV group. As a UAV network is a subclass of the mobile ad hoc network, the common attacks in the mobile ad hoc network will also threaten the UAV network.

Due to the diversity of network access methods and the openness of networks, UAV networks are facing inevitable security threats [9,10,11,12]. The defense function of traditional network security technology is mostly passive, and it is difficult to resist network attacks with changeable technology. As an active defensive network security technology, intrusion detection technology makes up for the shortcomings of traditional security technology [13,14,15,16].

While intrusion detection systems have attracted much attention from users, there are still some problems to be improved in their practical application. Traditional intrusion detection systems generally suffer from an insufficient performance and inefficiency, especially in modern computer networks with high bandwidth and large traffic. In the face of attacks, which are becoming more and more complex, automated, and distributed, traditional intrusion detection systems cannot meet the needs of current network security. In order to improve the detection efficiency and reduce the false alarm rate of intrusion detection systems, more and more researchers have introduced machine learning algorithms into the field of intrusion detection and have made good progress [17,18,19,20,21].

Shah et al. [22] investigated the performance of two open-source intrusion detection systems, Snort and Surcata. The results show that using an optimized support vector machine (SVM) and firefly algorithm can achieve the best detection effect. Kabir et al. [23] proposed a new method based on a least-squares support vector machine (LS-SVM) for an intrusion detection system. Wang et al. [24] proposed an intrusion detection framework based on the SVM, with feature augmentation. By transforming the logarithmic marginal density ratio to form original features, new and better transform features can be obtained, which greatly improves the detection ability of the model. Ahmed et al. [25] proposed a learning algorithm for an intrusion detection system based on a neural network (NN), which has a good performance in terms of its convergence speed and learning time. Hu et al. [26] proposed a distributed intrusion detection framework, in which a local parameterized detection model is constructed in each node using the online Adaboost algorithm. Ma et al. [27] proposed a novel approach called SCDNN, which combines spectral clustering (SC) and deep neural network (DNN) algorithms. The experimental results indicate that the SCDNN classifier performs better than the back-propagation neural network and support vector machine.

However, with the deepening of research, deep learning has gained wider application and a more outstanding performance in massive data analysis, which can be used to solve intrusion detection problems of massive, high-dimensional, and nonlinear data. By constructing a nonlinear network structure with multiple hidden layers, low-dimensional features, which are easier to classify in the data, can be obtained, and the accuracy of intrusion detection is improved [28,29,30,31,32]. Hinton et al. [33] proposed a deep learning method, called a deep belief network, which has attracted wide attention in academic circles. The deep belief network can transform high-dimensional and nonlinear data features into abstract features, which are more suitable for pattern classification, through layer-by-layer feature extraction. Qu et al. [34] proposed an intrusion detection model based on a deep belief network, which effectively improves the detection of abnormal data. Liang et al. [35] proposed an intrusion detection method based on a deep belief network and extreme learning machine, which improves the recognition rate of intrusion detection and the efficiency of the algorithm operation.

The number of nodes in the hidden layer of a deep belief network is not easy to determine. In this paper, the particle swarm optimization (PSO) algorithm is used to find the optimal number of hidden layer nodes. Common intelligent search algorithms include the genetic algorithm [36,37], ant colony algorithm [38,39], simulated annealing algorithm [40], and particle swarm optimization. The genetic algorithm cannot effectively converge in a limited time. The ant colony algorithm is slow in terms of its solving time and is prone to prematurity. The actual effect of the simulated annealing algorithm is greatly affected by the parameters, including the global optimization and calculation efficiency. The Bayesian optimization algorithm [41] is also commonly used for hyperparameter optimization. Its advantage is that it has fewer iterations, but it is easy to fall into local optimization. The PSO algorithm is easy to understand, easy to implement, fast in convergence, and can obtain the global optimal solution. Therefore, the PSO algorithm is selected as the optimization algorithm. The PSO algorithm is a kind of evolution algorithm based on a population. Through individual cooperation and group sharing, particles find the optimal solution of individuals and the optimal solution of the whole community to complete the optimization. Aburomman et al. [42] proposed a novel ensemble construction method that uses PSO-generated weights to create an ensemble of classifiers, which has a better accuracy in intrusion detection. Bamakan et al. [43] proposed an attack detection method based on multi-criteria linear programming and the PSO algorithm to improve the accuracy of attack detection.

In this paper, the deep learning method of the deep belief network (DBN) and the parameter optimization method of the PSO are introduced into the field of intrusion detection, and an intrusion detection model based on the PSO-DBN is proposed. The model uses the PSO algorithm to optimize the number of nodes in the DBN hidden layer to obtain the optimal network structure. Then, each restricted Boltzmann machine (RBM) network is trained from the bottom to the top, and the low-dimensional representation of the original data is obtained in the unsupervised learning process, which significantly reduces the dimensionality of the data, retains key features of the data, and removes the redundancy features. Finally, the back-propagation (BP) algorithm is used to classify the low-dimensional representation and fine-tune the RBM network at the same time. The PSO-DBN method, proposed in this paper, is compared to the artificial neural network (ANN), SVM, Adaboost, and DNN methods using the KDD Cup 99 dataset [44]. The experimental results show that the optimization effect of PSO is better than those of the genetic algorithm, simulated annealing algorithm, and Bayesian optimization algorithm, and the PSO-DBN model is superior to other machine learning methods, which effectively improves the accuracy of intrusion detection.

The remaining sections of this paper are organized as follows. Section 2 describes the principle of the DBN. Section 3 describes the parameter optimization based on the PSO algorithm. Section 4 describes the intrusion detection based on the PSO-DBN. Section 5 describes the experimental results and discussion, including the dataset, evaluation indicators, results, and comparison. Finally, Section 6 summarizes the paper.

## 2. Principle of the DBN

**Figure 1:**
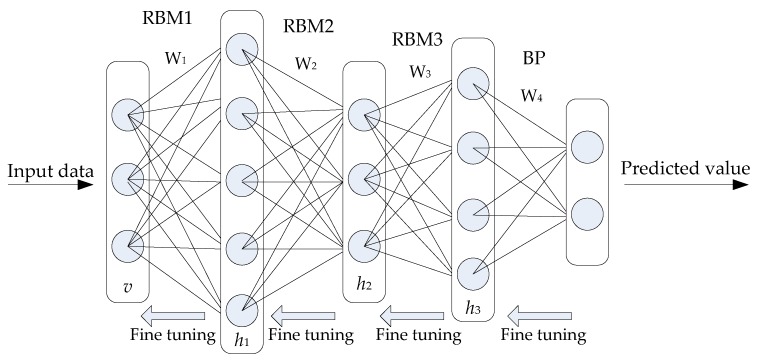
Detection model based on the deep belief network (DBN).

The detection model based on the DBN method is shown in Figure 1. The input layer includes five types of network data, including the Normal, Probing, DoS, U2R, and R2L data. A DBN is a neural network model, composed of multiple RBMs. When applying a DBN network in intrusion detection, the network structure should be trained first, to determine the connection weight and neuron bias of the network. The DBN mainly includes pre-training and reverse fine-tuning in the process of training the model. First, each layer of the RBM network is trained independently and unsupervised in the pre-training process to ensure that as much feature information as possible is retained when the feature vectors are mapped to different feature spaces. Then, the BP network is set-up in the last layer of the DBN, and the output eigenvector of the RBM is received as its input eigenvector. Then, supervised training is conducted for the entity relationship classifier. Moreover, each layer of the RBM network can only ensure that the weights in its own layer are optimal for the feature vector mapping of that layer, not for the whole DBN. Therefore, it is necessary for the BP network to spread error information, from top to bottom, in each layer of the RBM and fine-tune the DBN network. The process of training the model of the RBM network can be regarded as the initialization of the weights of a deep BP network, which makes the DBN overcome the shortcomings of the BP network, which easily falls into the local optimum due to the random initialization of weights.

A single RBM is a neural network model consisting of a visible layer and a hidden layer [45]. Figure 1 shows a network structure consisting of 3 layers of the RBM, where v is the visible layer connecting the intrusion detection data, h is the hidden layer, which is used to extract the effective features of the input data, and W is the connection weight of the visible layer and the hidden layer. The neurons of the same layer in the network structure are not connected to each other, and the neurons of the adjacent layers are connected to each other by weights. The inactivated and activated states are represented by a binary, 0 and 1, for neurons in the network.

The RBM is an energy-based model [46], where $v_{i}$ is used to represent the state of neuron i in the visible layer, with corresponding bias $a_{i}$, $h_{j}$ is used to represent the state of neuron j in the hidden layer, with corresponding bias $b_{j}$, and the connection weight of neuron i and j is $w_{ij}$. The energy of the RBM can be expressed as

(1) $$E\left(v,h|\theta \right)=-\sum_{i=1}^{n}a_{i}v_{i}-\sum_{j=1}^{m}b_{j}h_{j}-\sum_{i=1}^{n}\sum_{j=1}^{m}v_{i}w_{ij}h_{j}.$$

In the equation, $\theta =\left(w_{ij},a_{i},b_{j}\right)$ is the RBM parameter, and n and m are the number of neurons in the visible layer and hidden layer, respectively.

From the energy function, the joint probability distribution of (v,h) can be obtained as follows:

(2) $$p\left(v,h|\theta \right)=\frac{1}{Z\left(\theta \right)}\exp\left(-E\left(v,h|\theta \right)\right),$$

where $Z\left(\theta \right)=\sum_{v}\sum_{h}\exp\left(-E\left(v,h|\theta \right)\right)$ is the normalization factor.

For the training sample with the number of N, parameter θ is obtained by learning the maximum logarithmic likelihood function of the sample, which is

(3) $$\theta^{*}=\underset{\theta }{\arg\max}L\left(\theta \right)=\underset{\theta }{\arg\max}\sum_{n=1}^{N}\log p\left(v^{n}|\theta \right),$$

where $p\left(v|\theta \right)=\frac{1}{Z\left(\theta \right)}\sum_{h}\exp\left(-E\left(v,h|\theta \right)\right)$ is the likelihood function of v.

In the process of training, due to the complexity of calculating the normalization factor Z(θ), Gibbs and other sampling methods are generally used to approximate it [47]. Hinton proposed a fast learning algorithm using contrast divergence (CD) to train the network parameters, which improves the training efficiency and promotes the development of the RBM. The CD algorithm calculates the state of the neurons in the hidden layer by the vector value of the neurons in the visible layer, and then reconstructs the state of the neurons in the visible layer using the neurons in the hidden layer and calculates the state of the neurons in the hidden layer again using the reconstructed neurons in the visible layer, so that a new state of the neurons in the hidden layer can be obtained.

As the activation states of each neuron in the same layer of the RBM are independent of each other, the *j*th neuron in the hidden layer is calculated according to the state of the neurons in the visible layer, and the activation probability is as follows:

(4) $$p\left(h_{j}=1|v,\theta \right)=\sigma \left(b_{j}+\sum_{i}v_{i}w_{ij}\right),$$

where $\sigma =\frac{1}{1+\exp\left(-x\right)}$ is the sigmoid activation function.

The *i*th neuron in the visible layer is reconstructed by the hidden layer, and the activation probability is as follows:

(5) $$p\left(v_{i}=1|h,\theta \right)=\sigma \left(a_{i}+\sum_{j}w_{ij}h_{j}\right).$$

Further, the updated equations of the RBM weights and bias parameters can be obtained as follows:

(6) $$\left\{ \begin{array}{l} w_{ij}^{k+1}=w_{ij}^{k}+\varepsilon \left({\langle v_{i}h_{j}\rangle }_{data}-{\langle v_{i}h_{j}\rangle }_{recon}\right)\\ a_{i}^{k+1}=a_{i}^{k}+\varepsilon \left({\langle v_{i}\rangle }_{data}-{\langle v_{i}\rangle }_{recon}\right)\\ b_{j}^{k+1}=b_{j}^{k}+\varepsilon \left({\langle h_{j}\rangle }_{data}-{\langle h_{j}\rangle }_{recon}\right) \end{array}\right..$$

Among them, ${\langle \cdot \rangle }_{data}$ is the distribution, defined by the model of the original intrusion detection data, ${\langle \cdot \rangle }_{recon}$ is the distribution defined by the reconstructed model, ε is the learning rate, k is the number of iterations of the CD algorithm, $w_{ij}^{k+1}$ is the updated weight matrix, and $a_{i}^{k+1}$ and $b_{j}^{k+1}$ are the bias vectors, after the visible layer and the hidden layer have been updated.

## 3. Parameter Optimization Based on the PSO Algorithm

The PSO algorithm is inspired by the behavioral characteristics of bird predation and is used to solve the optimization problem. Each particle in the algorithm represents a potential solution to the problem, and each particle corresponds to a fitness value, which is determined by the fitness function. The velocity of the particle determines the direction and distance of the particle movement. The velocity is dynamically adjusted to the movement experience of the particle itself and other particles, thus realizing the optimization of the individual in the solvable space [48].

The PSO algorithm first initializes a group of particles in the solvable space, and in each iteration, the particles update themselves by tracking two extreme values. One is the optimal solution found by the particle itself, which is generally called the individual extreme value; the other is the current optimal solution, found by the whole population, which is generally called the global extreme value. The individual extreme value and global extreme value are updated continuously in the iteration process, and the final output global extreme value is the optimal solution, obtained by the algorithm [49].

It is supposed that in a D-dimensional search space, the population consisting of n particles is $X=\left(X_{1},X_{2},\cdots ,X_{n}\right)$, where the *i*th particle represents a D-dimensional vector, $X_{i}={\left(x_{i1},x_{i2},\cdots ,x_{it}\right)}^{T}$, which represents the position of the *i*th particle in the D-dimensional search space and also a potential solution to the problem. According to the fitness function, the fitness value corresponding to the position of each particle can be calculated. The fitness function defined in this paper is as follows:

(7) $$F_{fitness}=1-correct/sum,$$

where correct represents the number of data that are correctly classified, and sum represents the total number of data.

Assuming that the velocity of the *i*th particle is $V_{i}={\left(V_{i1},V_{i2},\cdots ,V_{iD}\right)}^{T}$, its individual extreme value is $P_{i}={\left(P_{i1},P_{i2},\cdots ,P_{iD}\right)}^{T}$, and the global extreme value of the population is $P_{g}={\left(P_{g1},P_{g2},\cdots ,P_{gD}\right)}^{T}$. In each iteration, the particle updates its velocity and position through the individual and global extreme value. The updating equation is as follows:

(8) $$V_{id}^{k+1}=\omega V_{id}^{k}+c_{1}r_{1}\left(P_{id}^{k}-X_{id}^{k}\right)+c_{2}r_{2}\left(P_{gd}^{k}-X_{id}^{k}\right),$$

(9) $$X_{id}^{k+1}=X_{id}^{k}+V_{id}^{k+1},$$

where d represents the *d*th dimension of the variable, d=1,2,⋯,D; i represents the *i*th particle, i=1,2,⋯,n; k is the current number of iterations; $V_{id}$ is the velocity of the *d*th dimension of the *k*th iteration of particle i; $P_{id}^{k}$ is the coordinates of the individual optimal value, found by particle i in the *d*th dimension of the *k*th iteration; $P_{gd}^{k}$ is the position of the global optimal solution, found by the entire particle swarm in the *d*th dimension of the *k*th iteration; $c_{1}$ and $c_{2}$ are learning factors, which are used to adjust the maximum step size for the optimal position of the individual and the optimal position of the group; $r_{1}$ and $r_{2}$ are random numbers distributed between [0, 1], called inertia factors, and the larger the value, the larger the range of the search; and ω is the inertia weight, which is a parameter introduced to balance the global search ability and local search ability. In order to prevent a blind search of particles, it is generally recommended to limit their position and velocity to a certain interval: $\left[-X_{\max},X_{\max}\right]$ and $\left[-V_{\max},V_{\max}\right]$.

## 4. Intrusion Detection Based on the PSO-DBN

**Figure 2:**
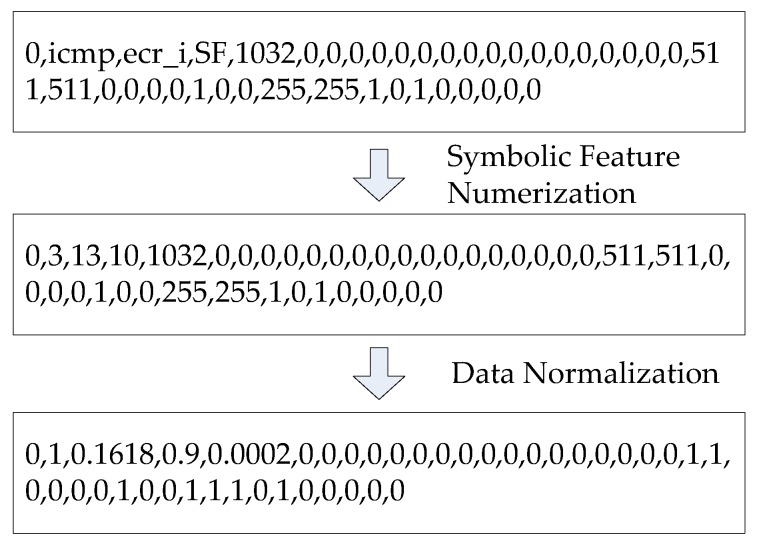
Preprocessing process of the connection record.

UAV mobile ad hoc network intrusion detection can be regarded as a classification problem. First, the intrusion detection dataset is preprocessed. The preprocessing process is shown in Figure 2. Each connection record in the KDD Cup 99 dataset consists of 41 attribute features, including 3 symbolic features and 38 numeric features. In this paper, the attribute mapping method is used to transform symbolic features into numeric features. For example, there are three values for the attribute feature, ‘protocol type,’ in column 2: tcp, udp, and icmp, which can be processed according to tcp = 1, udp = 2, and icmp = 3. Similarly, the 70 symbol values of the attribute feature, ‘service,’ and the 11 symbol values of the ‘flag’ can establish the mapping relationship between the symbol value and the corresponding numerical value.

Then, the obtained data are normalized, and the data are normalized within a range of [0, 1], according to Equation (10), to ensure that the attributes are within the same order of magnitude.

(10) $$\tilde{x}\left(i\right)=\frac{x\left(i\right)-x_{\min}}{x_{\max}-x_{\min}}.$$

In the equation, $\tilde{x}\left(i\right)$ is the normalized value of the input variable; x(i) is the original value of the input variable; and $x_{\max}$ and $x_{\min}$ are the maximum and minimum values of the original data, respectively.

After preprocessing the intrusion detection data, the DBN network structure is initialized, and then the PSO algorithm is used to optimize the number of nodes in each layer of the DBN hidden layer to obtain an optimal network structure. Common hyperparameters in the DBN include the learning rate, the number of network layers, and the number of nodes in each layer. For the learning rate, it mainly controls the learning progress of the model. The larger the learning rate, the faster the learning speed. Generally speaking, users can intuitively set the optimal value of the learning rate by using experience values or other types of learning materials. For the number of network layers, the larger the number of layers, the more complicated the calculation. Compared to image processing, the dimension of the dataset used in this paper is not very high, and the selected network layers can meet the requirements of intrusion detection. In DBN, the selection of the number of nodes in each layer is very important. It not only has a great impact on the performance of the established DBN network model, but can also easily lead to “over fitting” in training if it is not properly selected. At present, the calculation formulas for determining the number of nodes in each layer proposed in most literatures are for the case of very large training samples, and the obtained results are not necessarily optimal. In fact, the number of nodes in each layer obtained by various calculation formulas greatly varies. In order to avoid “over fitting” during training as much as possible, and to ensure a high enough network performance and generalization ability, it is necessary to optimize the number of nodes in each layer.

**Figure 3:**
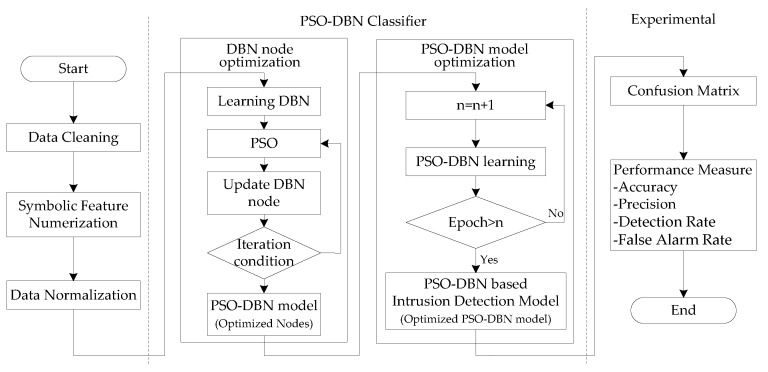
Intrusion detection model based on the particle swarm optimization (PSO)-DBN.

The intrusion detection model based on the PSO-DBN is shown in Figure 3. The process of building the DBN includes pre-training and reverse fine-tuning. First, the forward propagation of the DBN is established through the training of the RBM model, and better initial model parameters are obtained. Then, the output error information of the training samples is calculated by the BP algorithm and propagated, from top to bottom, in each layer of the RBM, and the parameters of the DBN model are finely adjusted.

In the process of optimizing the number of nodes in the hidden layer, the prediction error of the classifier is selected as the fitness function of the model. Through the iteration condition of the PSO, the number of nodes in the hidden layer of the DBN is constantly updated, and the optimized PSO-DBN model is obtained. When the PSO-DBN model is completed, supervised learning is performed using BP to obtain an improved performance in updating the values of the weights of the nodes. Therefore, learning is performed after assigning a suitable number of epochs of the BP.

## 5. Experimental Results and Discussion

### 5.1. Dataset and Evaluation Indicators

This paper uses the KDD Cup 99 dataset as the training and testing set. The dataset is derived from the intrusion detection assessment program of the US Department of Defense Advanced Research Projects Agency (DARPA). It is hosted by the MIT Lincoln Laboratory. It is the benchmark dataset of network intrusion detection. It provides labeled training data and test data for researchers and is widely used for testing various intrusion detection methods. In this paper, 10% of the data was randomly selected from the “10% KDD Cup 99 training set,” as the training data, and 10% of the data was randomly selected from the “KDD Cup 99 corrected labeled test data set,” as the test data. The specific data distribution is shown in Table 1.

In the intrusion detection system, the accuracy (ACC), precision (PRE), detection rate (DR), and false alarm rate (FAR) are usually used as indicators to evaluate the system. ACC is the proportion of correctly classified samples and is defined as follows:

(11) $$ACC=\frac{TP+TN}{TP+TN+FN+FP},$$

where TP refers to the number of positive instances detected as positive instances, TN refers to the number of negative instances detected as negative instances, FP refers to the number of negative instances detected as positive instances, and FN refers to the number of positive instances detected as negative instances.

PRE is the proportion of samples that have an actual intrusion behavior in the samples detected as intrusion behavior, and it is defined as

(12) $$PRE=\frac{TP}{TP+FP}.$$

DR is the proportion of the number of detected intrusion samples in the total number of intrusion samples, and it is defined as

(13) $$DR=\frac{TP}{TP+FN}.$$

FAR is the proportion of the number of normal samples that are falsely reported as intrusions in the total number of normal samples, and it is defined as

(14) $$FAR=\frac{FP}{TN+FP}.$$

The average reconstruction error (ARE) between the reconstructed data and the original data in each RBM network is also used as the criterion for performance evaluation, and it is calculated as follows:

(15) $$ARE=\frac{\sum_{k=1}^{n}\sum_{i=1}^{41}{\left(v_{ki}-v_{ki}^{\prime }\right)}^{2}}{n},$$

where k is the sample number; $v_{ki}$ is the original data of the *k*th sample; $v_{ki}^{\prime }$ is the *k*th sample data after reconstruction; and n is the number of samples.

### 5.2. Results and Comparison

The experimental environment of this paper is based on MATLAB R2013a and the data mining software, Weka. Compared to image processing, the dimension of the KDD Cup 99 dataset is not very high, so a DBN structure with four hidden layers can satisfy the experimental requirements. In order to verify the superiority of the PSO-DBN algorithm, proposed in this paper, a comparative experiment is carried out using the ANN, SVM, DNN, and Adaboost algorithms. The parameters of the PSO algorithm are shown in Table 2.

**Figure 4:**
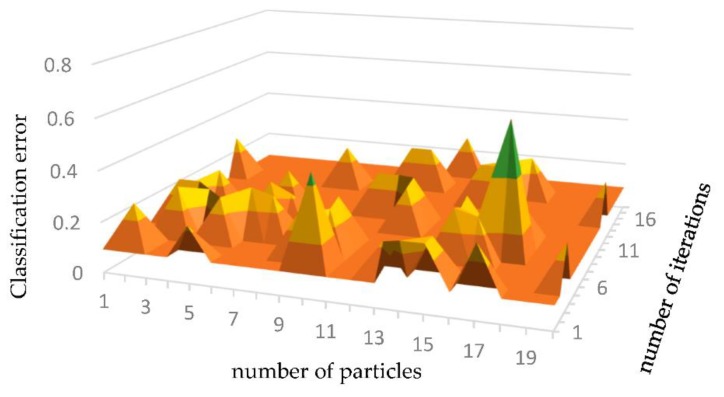
Optimization results of the PSO algorithm.

Figure 4 shows the results of the PSO algorithm for optimizing the number of hidden layer nodes under different iterations. In this paper, the classification error is used as the fitness function. In the process of PSO optimization, when the classification error is at its minimum, the optimal result can be obtained. The experimental results show that the numbers of hidden layer nodes optimized by the PSO algorithm are 39, 29, 14, and 7, and the minimum error is 0.0923.

**Figure 5:**
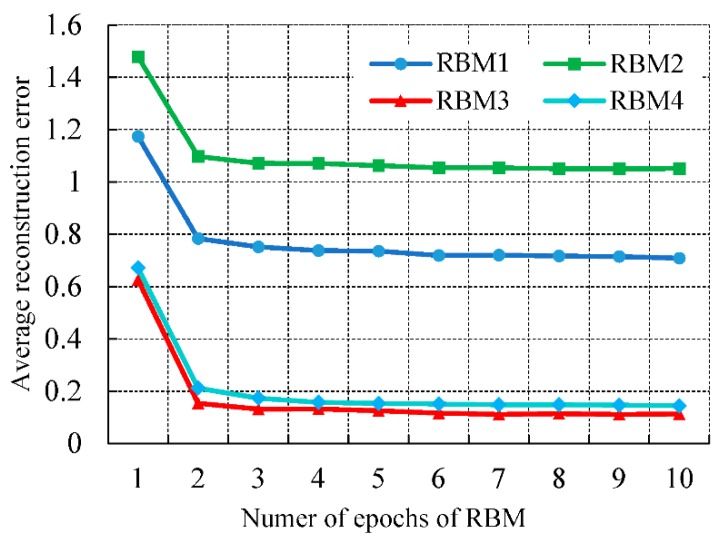
Average reconstruction error of the restricted Boltzmann machine (RBM).

The average reconstruction error of the RBM is shown in Figure 5. As can be seen from the figure, the higher the number of iterations of the RBM, the smaller the average reconstruction error. When the number of iterations is more than 4, the average reconstruction error tends to be flat.

**Figure 6:**
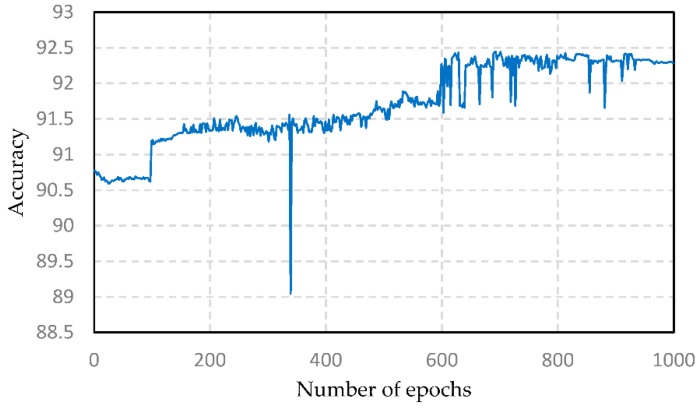
Accuracy under different BP epochs.

The accuracy under different BP epochs in the PSO-DBN model is shown in Figure 6. It can be seen, from the figure, that when the number of epochs is small, the accuracy of the proposed model increases with the increase in the number of BP iterations. When the number of epochs is 37, the accuracy of the model reaches a maximum of 92.44%. After that, the accuracy of the model decreases with the increase in the number of epochs and tends to be flat. Therefore, when the number of BP epochs is 693, the effect of the model is optimal.

The PSO-DBN model, proposed in this paper, is compared to the ANN, SVM, Adaboost, and DNN classification methods. The ANN algorithm uses a three-layer structure, including an input layer, an intermediate layer, and an output layer. The other parameters are similar to those of the DBN. The type of SVM algorithm is set to C-support vector classification (C-SVC), and the kernel function is the radial basis function. The weight threshold of the Adaboost algorithm is set to 100, and the number of iterations is set to 10. The DNN algorithm uses an eight-layer structure, including an input layer, six intermediate layers, and an output layer. The performance comparison of ANN, SVM, Adaboost, DNN, and PSO-DBN is shown in Table 3. It can be seen from the table that in dealing with the classification problem of intrusion detection, PSO-DBN has the lowest false alarm rate, the highest accuracy rate, detection rate, and precision, and the best classification effect.

For the optimization problem in this paper, comparative experiments of the genetic algorithm (GA), simulated annealing algorithm (SA), and Bayesian optimization algorithm (BOA) are carried out. The genetic algorithm is a kind of computing model that simulates natural selection and the genetic mechanism. It is an algorithm that finds the optimal solution by simulating the natural evolution process. The simulated annealing algorithm imitates the behavior of the burning object during the annealing process to find the optimal solution. The actual effect of the algorithm is greatly affected by the parameters. The Bayesian optimization algorithm finds an acceptable extreme value by guessing what the black box function (objective function) looks like without knowing what the black box function looks like. The advantage of the Bayesian optimization algorithm is that it has fewer iterations, but it is easy to fall into the local optimum. The parameters of the GA and SA are shown in Table 4 and Table 5, and the description of the BOA is shown in Table 6.

Table 7 shows the optimized number of nodes in each hidden layer. According to the optimized number of nodes in each hidden layer, the classification effect of the DBN network can be obtained. Table 8 shows the results of intrusion detection under various optimization algorithms. As can be seen from the table, the DBN network optimized by PSO has the lowest false alarm rate and the highest accuracy, detection rate, and precision. Therefore, the optimization effect of PSO is the best among the four optimization algorithms.

## 6. Conclusions

Intrusion detection for UAV networks is an important subject in the field of the security of UAV networks. The deep belief network optimized by PSO is a very effective method. Through the unsupervised learning of the RBM and the supervised learning of the BP, the DBN can effectively solve the intrusion detection problem of massive, high-dimensional, and nonlinear data. The DBN not only has a strong feature extraction ability for high-dimensional feature vectors, but it also has an efficient classification ability. Based on the DBN method, the PSO algorithm is used to optimize the number of hidden layer nodes of the DBN, to optimize its network structure. The experimental results show that the accuracy of the PSO-DBN algorithm, proposed in this paper, reaches 92.44%, which is higher than those achieved by the methods of ANN, SVM, Adaboost, and DNN. In addition, the optimization effect of PSO is better than those of GA, SA, and BOA. The PSO-DBN algorithm is very suitable for the tasks of information extraction in high-dimensional space, improves the intrusion recognition ability, and provides an effective solution to the problem of intrusion detection.

## Figures and Tables

**Table 1:** Distribution of various types of data in the dataset.

Types\Distribution	Training Data	Testing Data	Label
Normal	9626	6047	1
Probing	452	412	2
DoS	39216	23037	3
U2R	9	18	4
R2L	97	1586	5

**Table 2:** Parameters of the PSO algorithm.

Parameter Name	Value	Description
inertia weight	1	the recommended global and local convergence speed
acceleration factor	1, 1	c1 + c2 ≤ 4
iterations	20	if more than 20, the experimental results have no significant changes
population	20	the number of particles involved in the search at the same time
particle dimension	4	The number of hidden layers is 4

**Table 3:** Performance comparison of ANN, support vector machine (SVM), Adaboost, deep neural network (DNN), and PSO-DBN.

Result\Methods	ANN	SVM	Adaboost	DNN	PSO-DBN
ACC	90.79%	83.97%	90.00%	91.36%	92.44%
DR	89.68%	80.63%	89.00%	89.71%	91.20%
PRE	99.61%	99.54%	99.35%	99.60%	99.82%
FAR	1.46%	1.54%	2.40%	1.49%	0.68%

**Table 4:** Parameters of the GA.

Number of Generation	Population Size	Mutation Rate	Crossover Rate
20	10	0.05	0.8

**Table 5:** Parameters of the SA.

Decay Scale	Step Factor	Start Temperature	Final Temperature
0.85	0.2	8	3

**Table 6:** Description of the Bayesian optimization algorithm (BOA).

Prior Function	Acquisition Function	Number of Iteration	Objective Function
Gaussian process Regression	EI Function	30	Error in Classification

**Table 7:** Number of nodes in each hidden layer after optimization.

Hidden Layer Nodes\Methods	GA	SA	BOA	PSO
Layer 1	36	35	35	39
Layer 2	21	33	29	29
Layer 3	16	21	16	14
Layer 4	11	10	6	7

**Table 8:** Performance comparison of GA-DBN, SA-DBN, BOA-DBN, and PSO-DBN.

Result\Methods	GA-DBN	SA-DBN	BOA-DBN	PSO-DBN
ACC	91.66%	91.41%	91.30%	92.44%
DR	90.42%	90.22%	90.70%	91.20%
PRE	99.50%	99.50%	99.76%	99.82%
FAR	1.87%	1.89%	0.89%	0.68%
